# Occlusive dressings for fingertip amputations: Clinical outcomes, pulp regeneration, and dermatoglyphic recovery

**DOI:** 10.1016/j.jpra.2025.12.004

**Published:** 2025-12-12

**Authors:** Paul Zaoui, Maurice Renom, Francois Loisel, Soline Vericel, Laurent Obert, Isabelle Pluvy

**Affiliations:** Department of Orthopedics, Traumatology and Plastic Surgery, SINERGIES Laboratory, Marie and Louis Pasteur University, Besançon University Hospital (CHU Besançon), Besançon F- 25000, France

**Keywords:** Fingertip injury, Fingertip reconstruction, Occlusive dressing, Dermatoglyphs

## Abstract

**Objectives:**

To evaluate the clinical and functional outcomes of fingertip reconstruction with conservative treatment using occlusive dressings up to zone 3.

**Patients and methods:**

A retrospective monocentric study was conducted on 26 patients (28 fingers) who presented an amputation in zones 1, 2, or 3, treated with occlusive dressings. The evaluation included epidemiological data on the trauma, the number of dressings required, healing time, satisfaction, recovery of sensation, regeneration of dermatoglyphs, pulp trophicity, complications, and functional scores.

**Results:**

At a mean follow-up of 11.8 months, healing was achieved in 4.2 weeks after an average of four dressings. Pulp trophicity was excellent or good in 96.4 % of cases. Finger mobility was fully preserved in 89.3 % of fingers. Complete or partial dermatoglyphic regeneration was observed in all patients. Sensitivity tested by Weber's test was reduced by 24 % (4.6 mm vs. 3.5 mm contralateral). Nail dystrophies were noted in 60.4 % of fingers, mainly following amputations in zone 3. Cold intolerance was reported in 35.7 % of fingers. Functional scores confirmed satisfactory recovery. Satisfaction was high. The most frequently reported inconvenience was odor occasionally reported despite the use of charcoal dressings.

**Conclusion:**

Occlusive dressings represent a reliable, non-invasive, and reproducible alternative in fingertip amputations in zones 1 and 2. Despite limitations in zone 3, the aesthetic and functional outcomes justify its use as a first-line treatment. Dermatoglyphic regeneration is a real phenomenon, with both functional and legal implications. These results support occlusive dressing as a first-line management option for selected fingertip amputations.

## Introduction

Fingertip amputations are a common emergency in hand surgery, often resulting from domestic or occupational accidents. These injuries can lead to significant functional, aesthetic, and socio- professional consequences, compromising fine sensation, grip, and body image.^1^

Management depends on several factors, including the level of amputation, patient characteristics (age, occupation, activities), and the surgeon’s preferences.^2^

The treatment aims to achieve two primary goals: optimal tissue coverage and restoration of essential fingertip functions such as fine sensitivity, high-quality grip through good skin trophicity, and a satisfactory aesthetic outcome.^3^

Historically, two main strategies have been used: surgical management using skin flaps (such as Atasoy, Hueston, or Venkataswami-Subramanian flaps) and directed wound healing.

While flaps provide immediate coverage for areas with bone exposure, they are associated with a non-negligible risk of complications such as necrosis, residual sensory disturbances, or joint stiffness.^4^

As an alternative, conservative treatment using occlusive dressings has gradually emerged as a simple, cost-effective, and non-invasive approach. This method relies on the principle of moist wound healing, which promotes spontaneous tissue regeneration, even in the presence of dermal involvement.^5^ It is mainly indicated for amputations located in zones 1 and 2 according to the Merle and Dautel classification, and occasionally in zone 3.^6^^,^^7^

Although several studies have reported satisfactory functional outcomes with this method— particularly regarding sensory recovery and the reappearance of dermatoglyphs—data remain limited, especially concerning more proximal injuries.^8^

The aim of this study is to evaluate, through a retrospective series of 28 cases, the clinical, functional, and aesthetic outcomes of conservative treatment with occlusive dressings for fingertip amputations, including those extending to zone 3. Our hypothesis is that this treatment enables rapid healing with good sensory recovery and high-quality pulp regeneration, without requiring surgical intervention.

## Materials and methods

A retrospective, single-center study was conducted between January 2023 and December 2024 in the Hand Surgery Department of the University Hospital (CHU) of Besançon. The study included a consecutive series of patients presenting with fingertip amputations treated exclusively with occlusive dressings.

Inclusion criteria were patients with a fingertip amputation, regardless of the trauma mechanism, involving zone 1, 2, or 3 according to the Merle and Dautel classification; initial exclusive conservative management using occlusive dressings; and complete clinical follow-up with a minimum post-treatment evaluation period of 4 months. Exclusion criteria included active infection at the time of trauma; known history of abnormal wound healing; or incomplete or lost follow-up.

**Figure 1:**
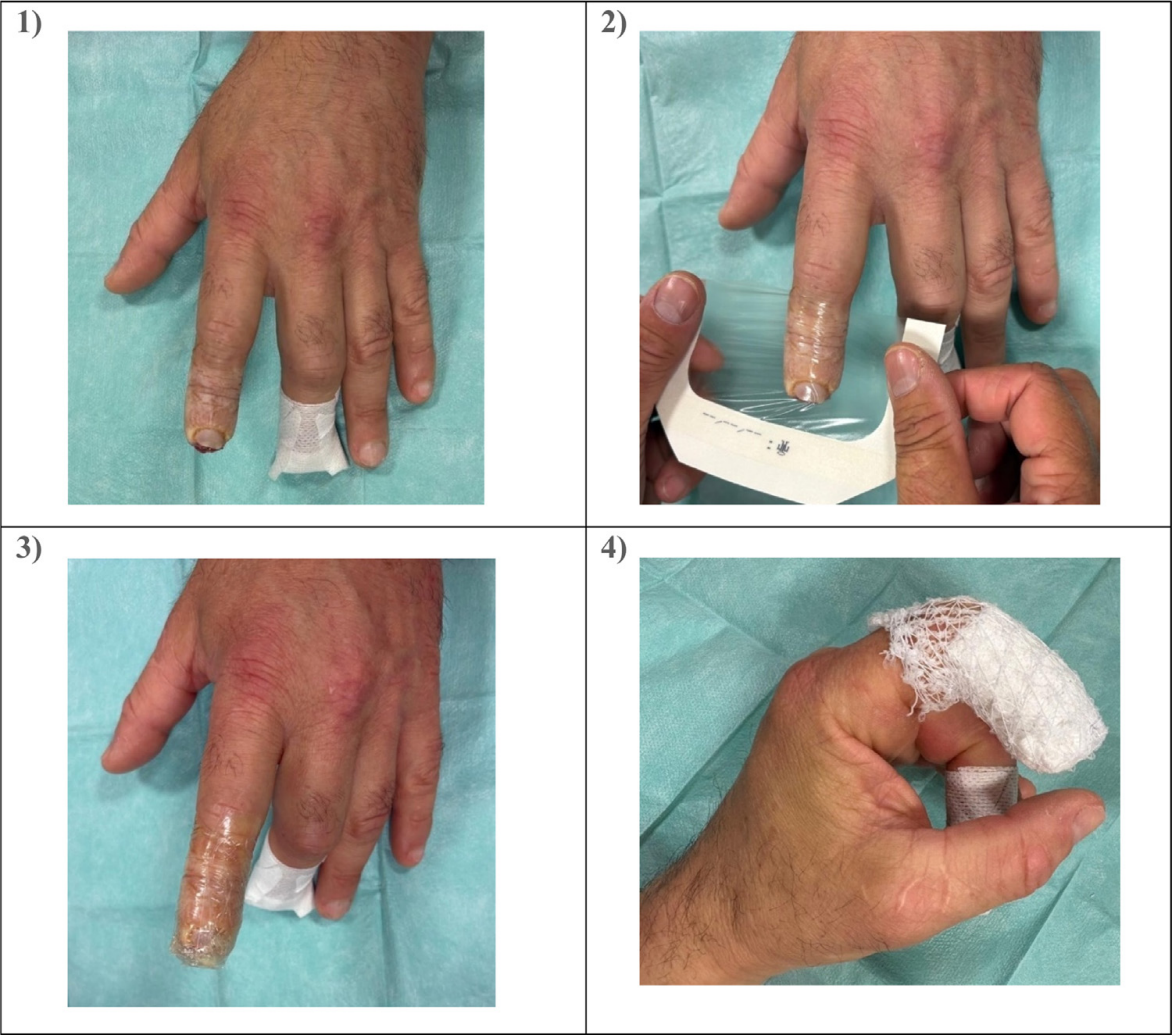
Application of an occlusive dressing: the same method was used at each dressing change. Ideally, the dressing should not cover the proximal interphalangeal joint to allow finger mobility.

Treatment was initiated on an emergency basis, after wound irrigation with saline and, if necessary, local anesthesia. The wounds were covered using two semi-permeable dressings (Tegaderm®, 3M™), wrapping the pulp up to the proximal interphalangeal joint while leaving it as free as possible to preserve joint mobility (Figure 1). No antiseptics or topical antibiotics were used. Dressings were changed weekly during outpatient visits until complete healing, defined as the presence of a stable, painless skin covering. Depending on the healing progression, three to six dressing changes were required. A foul odor was sometimes noted and managed by adding activated charcoal dressings.

Evaluations were performed by an independent examiner at least 5 months after complete healing. The study followed the ethical principles outlined in the Declaration of Helsinki.

This study was approved by the institutional review board of CHU Besançon. Collected data included:

- Demographic and injury-related data: age, sex, injured finger, trauma mechanism, amputation zone.
- Healing parameters: number of dressings used, time to complete healing, time to return to work.
- Pulp evaluation: Trophic quality of the fingertip pulp was assessed by direct comparison with the contralateral, uninjured finger. The evaluation relied on three predefined criteria: pulp volume, contour, and soft-tissue consistency.

• Excellent trophicity: Pulp volume strictly identical to the contralateral side.
• Normal fingertip contour with no visible depression, flattening, or asymmetry. Soft-tissue consistency comparable to normal pulp, with no areas of induration or atrophy.
• Good trophicity: Slight decrease in pulp volume (< 25 % compared with the contralateral side). Minor contour irregularities (mild flattening or slight asymmetry) without functional impact. Soft-tissue consistency near normal, with only minimal firmness or thinning.
• Poor trophicity: Significant loss of pulp volume (≥ 25 % compared with the contralateral side). Marked contour alterations (hollowing, visible tissue defect, prominent dorsal hook). Noticeable soft-tissue changes such as firmness, atrophy, or persistent tenderness.

This structured scoring system helped ensure greater reproducibility of the assessments between examiners.

- Sensory evaluation: two-point discrimination test (Weber test), monofilament testing (Touch-Test™ Sensory Evaluator).
- Complications: dysesthesia, cold intolerance, nail dystrophy, secondary infections.
- Functional outcomes: QuickDASH score (Appendix 1), FIOS score (Appendix 2).
- Articular function (Appendix 3): digital mobility test (maximum score = 11).
- Dermatoglyphic regeneration: assessed by comparing fingerprints.
- Patient satisfaction: recorded using a three-level subjective scale (very satisfied, satisfied, disappointed).

Quantitative data are presented as means and standard deviations. Qualitative variables are expressed as counts and percentages.

## Results

A total of 26 patients, corresponding to 28 fingers, were included in this study. All were treated with occlusive dressings for distal digital amputations between 2023 and 2024. The population was predominantly male (84.6 %), with a mean age of 47.3 years (range 17–72). The injury mechanisms included 13 clean cuts, 13 crush injuries with associated lacerations, and two mutilating injuries. Most amputations involved zones 1 and 2 (57.1 %) (Table 1). The mean follow-up time at the point of evaluation was 11.8 months (range 5–22).

**Table 1:** Patient characteristics.

Number of patients	26
Sex	four females, 22 males
Mean age[range]	47,3 years [17–72]
Injured digits	seven thumbs, six index fingers, five middle fingers, six ring fingers, four little fingers
Mechanism of injury	13 clean cuts, 13 crush + cut injuries, two mutilations
Amputation zone	three zone 1, 13 zone 2, 12 zone 3

**Figure 2:**
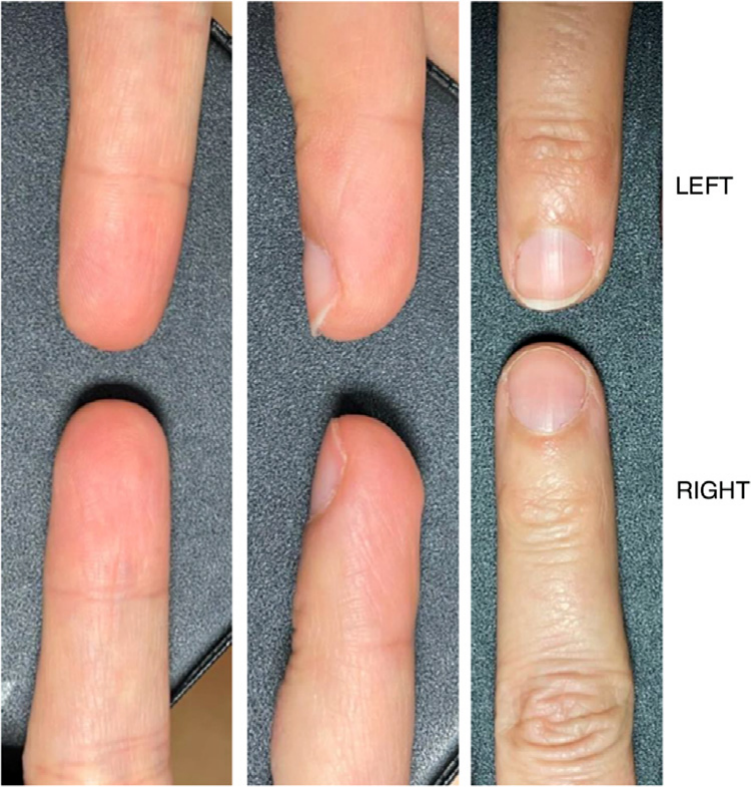
Comparison between healthy digit (left third finger) and injured digit (right third finger, zone 2 amputation) showing excellent pulp trophicity after three occlusive dressings.

**Figure 3:**
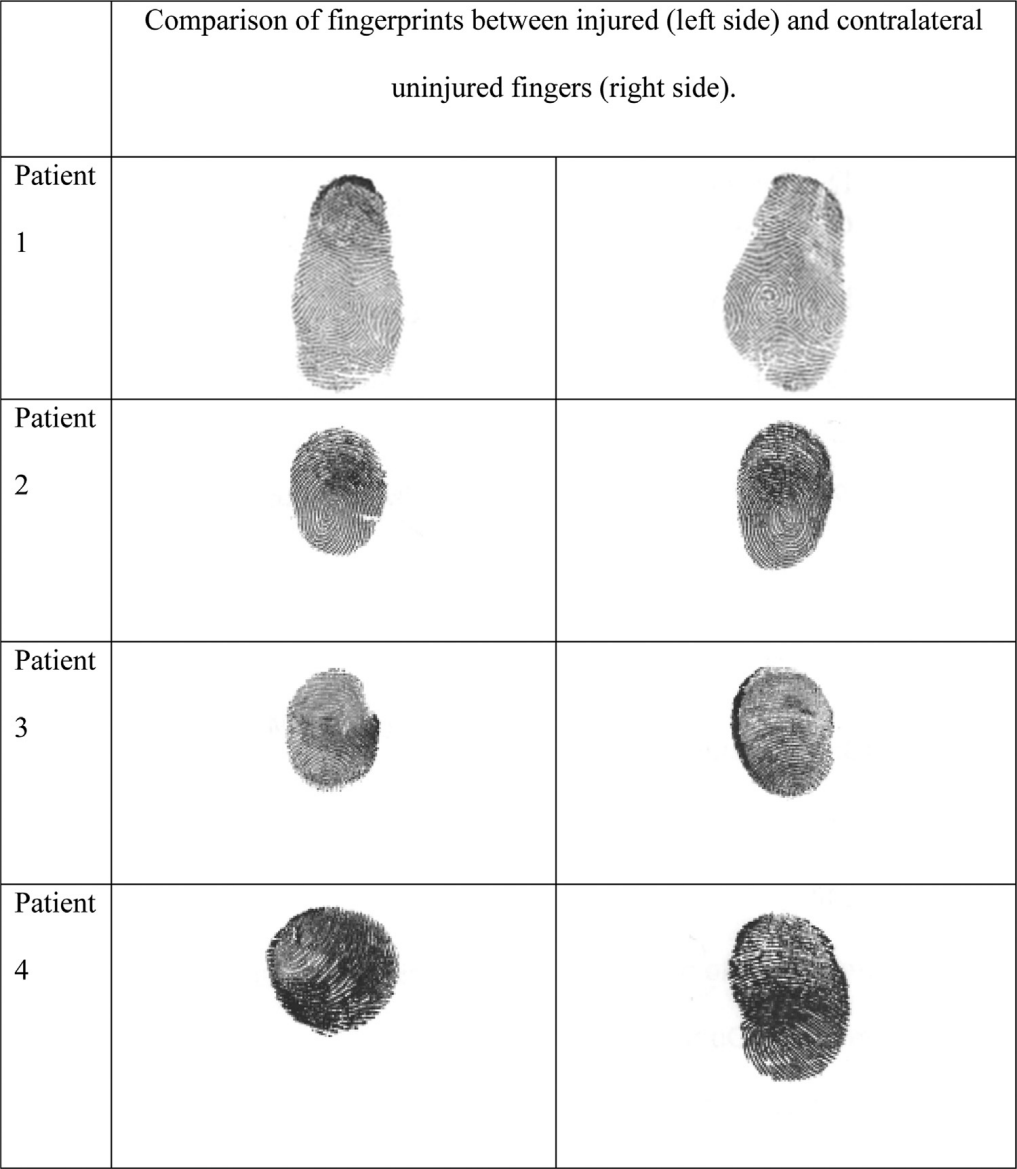
Dermatoglyphic regeneration on the injured finger in four patients at more than 1-year post-trauma.

The mean number of occlusive dressings per finger was four (range 1–7). Complete healing was achieved after an average of 4.2 weeks. Pulp trophicity was rated as excellent in 11 cases (39.3 %), good in 16 cases (57.1 %), and poor in one case (3.6 %) (Figure 2). Dermatoglyphic regeneration was observed in 100 % of cases, ranging from partial and coarse to complete and fine restoration (Figure 3).

Regarding sensitivity, the Weber two-point discrimination test showed an average of 4.6 mm on the injured side versus 3.5 mm on the healthy side, representing a 24 % decrease in sensitivity. Seventeen patients (65 %) exhibited normal sensitivity in the monofilament test. Fine sensory impairment was observed in six patients (21 %), protective sensitivity loss in one patient (3.5 %), and complete anesthesia in another (3.5 %). The mean monofilament perception threshold was 3.18 on the injured side versus 2.86 on the contralateral, uninjured side.

Twenty-five fingers exhibited full digital mobility, while three showed reduced mobility. The mean score on the digital mobility test was 10.43 out of 11. Persistent dysesthesia was reported in 12 patients (42.9 %), and cold intolerance in 10 patients (35.7 %).

**Figure 4:**
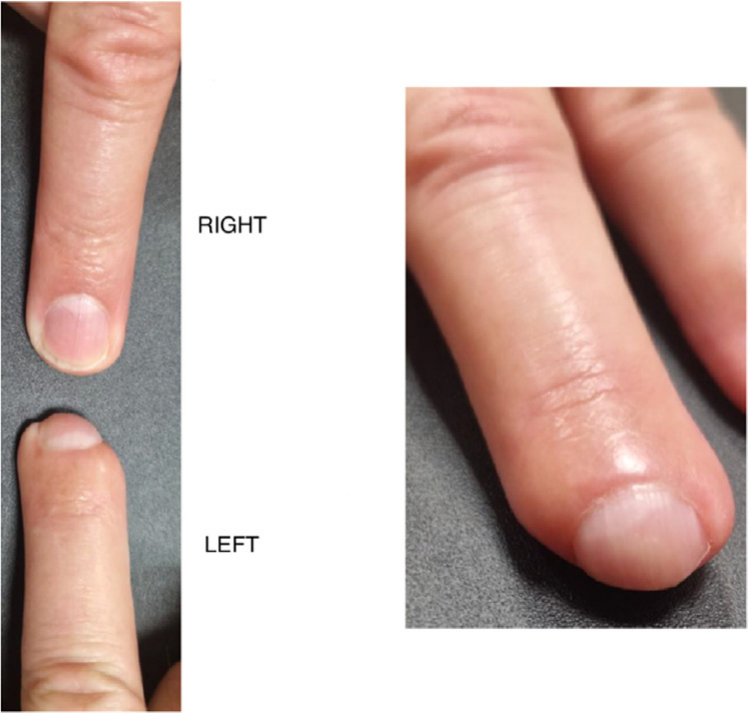
Comparison of healthy finger (right fourth finger) and injured finger (left fourth finger, zone 3 amputation) demonstrating claw nail deformity after five occlusive dressings.

Regarding complications, 15 patients presented with nail dystrophy (five cases of hook nail and 12 of wavy nail). These deformities, predominantly observed in zone 3, are illustrated in Figure 4. One case of paronychia during nail regrowth required surgical revision. Additionally, 10 patients (38.5 %) reported unpleasant odor from the dressings despite the use of activated charcoal.

At long-term follow-up, 19 patients (67.9 %) reported being very satisfied with the outcome, eight (28.6 %) satisfied, and one disappointed. Mean functional scores were: QuickDASH: 9.5 (range 0–63.6), FIOS: 11.96 (range 10–19).

## Discussion

This retrospective study of 26 patients (28 fingers) treated with occlusive dressings for distal digital amputation confirms the effectiveness of this conservative approach. Satisfactory pulp regeneration was achieved after a mean of four dressing changes, consistent with the literature, although results were more heterogeneous for zone 3 amputations (Table 2).

**Table 2:** Summary of characteristics and outcomes from major published series, along with our own, using occlusive dressings for fingertip amputations.

Study	Zone(s) treated	Mean no of dressings [min–max]	Complications	Healing time in weeks [min–max]	Healing outcome
Fox et al.^22^ 22 cases	1	4	0	4	Acceptable
Farell et al.^23^	1 (*n* = 15)	NR	two stiffness	Zone 1: 2	Acceptable
21 cases	Zone with bone exposure (*n* = 6)		four hypersensitivities	Zone with bone	
			three hypoesthesia	exposure: 4,3 to 6,4	
Louis et al.^24^	1	3,5	four cold intolerances + dysesthesia (13,8 %)	NR	NR
29 cases			three cold intolerances alone (10,3 %)		
De Boer et al.^25^	1 (*n* = 25)	NR	0	1,7	Good
29 cases	Zone with bon exposure (*n* = 4)				
Mennen et al.^26^	NR	2,5	0	[2,9–4,3]	Excellent
200 cases					
Quell et al.^27^	1 (*n* = 26)	[2–8]	0	[1–9,1]	Good
42 cases	2 (*n* = 8)				
Lasserre et al.^13^	3 (*n* = 8)				
19 cases	1 (*n* = 13)	3,8 [2–7]	0	3	Excellent 30 %
	2 (*n* = 5)				Good 70 %
	3 (*n* = 1)			[2–5]	
Hoigné et al.^6^	2 and 3	4	one hypersensitivity at 6 months	6,5	Good
19 cases			one neuroma	[3–8]	
			one nail dystrophy (5,2 %)		« 90 % pulp reconstruction »
Boudard et al.^5^	1 (*n* = 9)	3,2 [3–5]	four cold intolerances (21 %)	4,8	Excellent 32 %
	2 (*n* = 6)				Good 63 %
	3 (*n* = 4)		four nail dystrophy (21 %) including three claw nails		Poor 5 %
19 cases					
Pastor et al.^9^	NR	NR	15 cold intolerances (22,7 %)	< 6	Excellent
66 cases			two dysesthesia (3 %)		
			12 nail dystrophies (18,2 %)		
Bensa et al.^10^	2 (*n* = 18)	NR	20 cold intolerances (87 %)	4,9	Good 43 %
23 cases			three dysesthesia (13 %)		Fair 26 %
			eight nail dystrophies (34,8 %)		Poor 31 %
	3 (*n* = 5)		five adherent scars		
Our series	1 (*n* = 3)	4 [1–7]	10 cold intolerances + dysesthesia (35,7 %)	4,2	Excellent 40 %
	2 (*n* = 13)		15 nail dystrophies (53,6 %) including five claw nails,		Good 57 %
	3 (*n* = 12)		one paronychia		Poor 3 %

NR, Not reported.

To ensure consistency and reliability in the clinical evaluation, pulp trophicity and sensory outcomes were assessed according to predefined criteria established within the department. All examiners were specifically trained to apply these criteria uniformly. When circumstances allowed, assessments were cross-checked by a second examiner during follow-up visits to confirm the classification. In cases of discrepancy, the final rating was established through discussion. This approach helped reduce variability and strengthened the reproducibility of the evaluations within the study. Nonetheless no formal inter-observer reliability coefficient was calculated.

Functional recovery was generally good, particularly regarding sensory outcomes. The Weber test showed an average two-point discrimination of 4.6 mm on the injured side compared to 3.5 mm on the healthy side, results comparable to those reported in previous publications.^9^ In the literature, several studies have compared sensory recovery after surgical treatment using local flaps versus conservative management. Bensa et al. found no significant difference between the two groups,^10^ whereas Söderberg et al. reported better sensory recovery in favor of occlusive dressings in a comparative series of 70 fingertip amputations.^11^

The systematic regeneration of dermatoglyphics observed in our series is also a noteworthy finding, as fingerprints play a central role in the mechanical transduction of tactile stimuli.^12^ They also have significant functional and legal implications.

Occlusive dressings present several practical advantages. This method is simple to implement, minimally restrictive for the patient, and requires only weekly dressing changes. The polyurethane film acts as a second skin, reducing pain, promoting early mobilization, and thus preventing joint stiffness.^13^ The quality of wound healing is satisfactory, with pulp trophicity rated as excellent or good in >96 % of cases. Jafari et al. similarly reported a pulp thickness nearly identical between injured and uninjured fingers after conservative treatment.^14^

A comparison with other conservative approaches also helps contextualize our findings. Traditional secondary intention healing using simple dressings, paraffin gauze, or silver sulphadiazine requires more frequent dressing changes, provides less protection, and may increase patient discomfort. Semi-occlusive dressings, in contrast, maintain a stable and protected humid microenvironment with minimal manipulation, promoting faster epithelialization and improved pain control. Recent evidence further supports the relevance of conservative treatment for more severe fingertip injuries. Thai Van Nguyen and al. demonstrated that even in cases with exposed distal phalanx, directed wound healing can achieve satisfactory soft-tissue regeneration and functional outcomes.^15^ While secondary intention healing may be effective in selected cases with limited bone exposure, the quality and predictability of regeneration and pain control appear less consistent than with semi-occlusive polyurethane films. Together, these complementary findings reinforce the relevance of conservative strategies and suggest that spontaneous healing may represent a viable alternative to surgical coverage in selected fingertip or complex injuries.

Moreover, this approach is less costly: it requires neither surgery, hospitalization, nor home nursing care. Cheang et al. estimated a cost reduction of 60 % compared with surgical treatment.^16^

Nevertheless, this technique is not without drawbacks. An unpleasant odor due to maceration was reported by 38.5 % of patients in our series, despite the use of charcoal dressings.^17^ Some authors recommend specific interfaces to limit this effect.^18^ In addition, weekly follow-up is essential for an average duration of 3 to 4 weeks and may extend up to 7 weeks in some cases.^19^

Compared with surgery, this method avoids frequent complications such as joint stiffness, partial or total flap necrosis, delayed healing, or infection.^20^ However, sequelae such as cold intolerance or dysesthesia may persist regardless of the treatment technique. Söderberg et al. even reported a higher frequency of these symptoms in surgically treated groups,^11^ and several authors consider these manifestations to be more closely related to the initial trauma than to the therapeutic strategy itself.^21^

Infectious complications associated with occlusive dressings are regularly mentioned in the literature.^27^ However, no study has demonstrated an increased rate of infection in series treated conservatively, despite the sometimes-bothersome odor reported by patients.^28^ Bacteriological analyses consistently show polymicrobial colonization of the exudate, including aerobic and anaerobic organisms.^13^ This commensal flora may facilitate autolytic debridement without increasing infectious risk.^29^, ^30^

Nail dystrophies, particularly claw nails, are frequent complications of zone 3 amputations. This location therefore appears more prone to aesthetic and functional sequelae. The indication for occlusive dressings in these situations should be carefully evaluated. Bensa et al. reported a significantly higher incidence of claw nail deformities in the occlusive dressing group compared with the surgical group (60 % vs. 0 %) for zone 3 amputations.^10^

This observation, corroborated by the data of Muneuchi et al.^31^, suggests that the therapeutic strategy for pulp injuries should consider not only the injury zone, but also the orientation of the amputation (transverse, palmar oblique, dorsal oblique, lateral oblique). In cases of transverse amputation in zone 3, occlusive dressings may be considered, provided no bony prominence remains. For oblique amputations, a local flap is generally preferable to optimize functional and aesthetic results. However, the use of occlusive dressings should not be formally excluded in these cases.

The objectives of managing fingertip amputations are multiple: to ensure reliable soft-tissue coverage, restore functional sensation, preserve finger length, maintain joint mobility, achieve an acceptable cosmetic appearance, and ensure patient satisfaction. The results of our study confirm the ability of occlusive dressings to meet these requirements, particularly for injuries in zones 1 and 2.

In conclusion, although this technique presents certain limitations—particularly in zone 3—occlusive dressings represent a simple, effective, and minimally invasive alternative, with a low complication rate and satisfactory functional recovery. Wider adoption could help reduce the need for potentially unnecessary surgical procedures in the management of distal digital amputations. Furthermore, dermatoglyphic regeneration is a real phenomenon with both functional and legal implications.

## Conclusion

Occlusive dressings represent a reliable and effective therapeutic alternative for the management of digital pulp amputations in zones 1 and 2. They promote pulp tissue regeneration, with restoration of contour and reappearance of dermatoglyphics. After three to four dressing changes—approximately 1 month of treatment—both aesthetic and functional results are generally satisfactory. Pulp sensitivity recovery may be equivalent to, or even superior to, that observed after surgical treatment.

In contrast, for zone 3 amputations, this method has certain limitations, particularly an increased risk of nail dystrophy. In such cases, surgical intervention may still be necessary to achieve an optimal outcome.

Occlusive dressings also have the advantage of being simple to implement, without the need for prolonged hospitalization, and are associated with a low complication rate. Only transient olfactory discomfort has been reported during treatment.

While effective and well tolerated, this conservative treatment warrants further investigation, particularly to better understand the mechanisms involved in the regeneration of pulp structures and fingerprints.

## Funding

This research did not receive any specific grant from funding agencies in the public, commercial, or not-for-profit sectors.

## Informed consent

This study was performed in accordance with the ethical standards of the 1964 Declaration of Helsinki, and approved by the institutional review board of CHU Besançon (N° 2025/993). All participants provided informed consent for the use of their data. This article complies with the data protection and privacy policy for which the CNIL has certified that the Besançon University Hospital (CHUB) is in compliance with MR004.

## Declaration of generative AI and AI-assisted technologies in the writing process

The authors did not use any generative AI or AI-assisted technologies to write this manuscript.

## Declaration of competing interest

The authors declare that they have no competing financial interests for this article.

Francois Loisel: Consultant for Medartis. Other affiliations: Arthrex, Evolutis, ZimmerBiomet and Elsevier.

Laurent Obert: Consultant for Medartis, Evolutis, FX Solutions, Kérimédical, Branchet. Royalties from FX Solutions, Elsevier and Sauramps.

Zaoui Paul, Maurice Renom, Soline Vericel and Isabelle Pluvy: No conflicts of interests.

## Appendix Supplementary materials

Image, application 1
