# Supplementary material for: Occlusive dressings for fingertip amputations: Clinical outcomes, pulp regeneration, and dermatoglyphic recovery
Source: JPRAS Open. 2025 Dec 12;48:593–602. doi: 10.1016/j.jpra.2025.12.004 (PMC12818269; doi:10.1016/j.jpra.2025.12.004)
Supplement: Supplementary file 1 [file mmc1.docx]

**Appendices:**

**Appendix 1: QUICK DASH score**

**
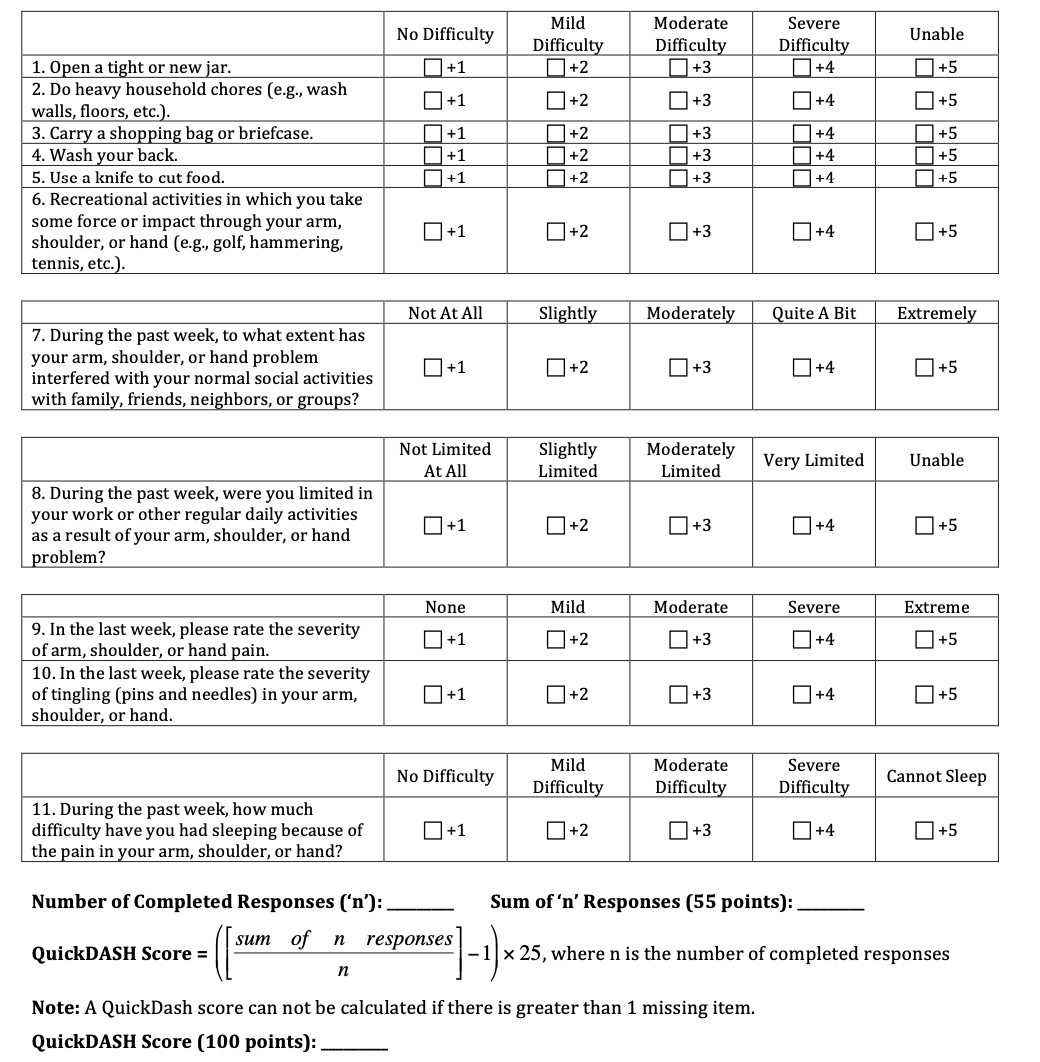
**

**Appendix 2: FIOS Score**

Fingertip Injuries Outcome Assessment Score

| **Criterion** | **Description** | **Score** |
| --- | --- | --- |
| Nail | Normal | 1 |
|  | Small nail | 2 |
|  | Split or deformed nail | 3 |
|  | Hooked nail | 4 |
|  | Absence of nail | 5 |
| Finger length | Distal third | 1 |
|  | Middle third | 2 |
|  | Proximal third | 3 |
| Pulp | Well-padded | 1 |
|  | Pulp atrophy | 2 |
| Bone | Consolidated fracture or normal | 1 |
|  | Pseudarthrosis | 2 |
|  | Bone shortening | 3 |
| Aesthetic appearance | Satisfactory | 1 |
|  | Unsatisfactory (poor colour match) | 2 |
| Sensation (2-PD) | <6 mm | 1 |
|  | 7–10 mm | 2 |
|  | Cold intolerance | 3 |
|  | No sensation / hyperalgesia | 4 |
| Pain | No pain | 1 |
|  | Mild | 2 |
|  | Moderate | 3 |
|  | Severe | 4 |
| Range of motion | 75%–100% | 1 |
|  | 50%–74% | 2 |
|  | <49% | 3 |
| Grip strength | 75%–100% | 1 |
|  | 50%–74% | 2 |
|  | <49% | 3 |
| Return to work | Regular work | 1 |
|  | Work with restrictions | 2 |
|  | Unable to work | 3 |

Evaluation Results

| Result | Score value range |
| --- | --- |
| Excellent | ≤12 |
| Good | 13–18 |
| Fair | 19–24 |
| Poor | >24 |


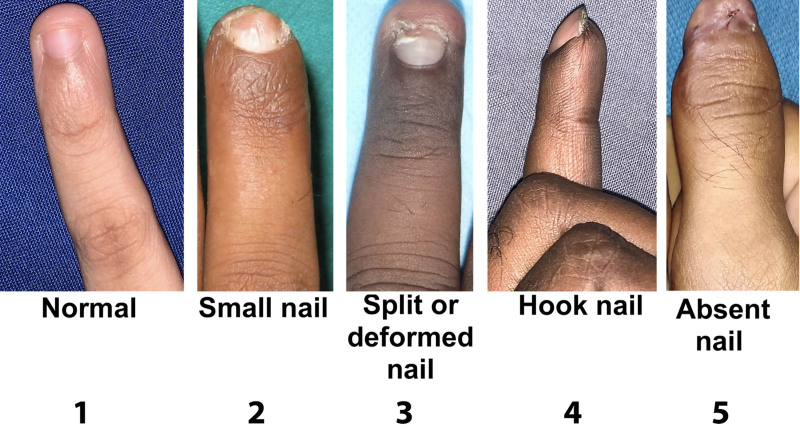

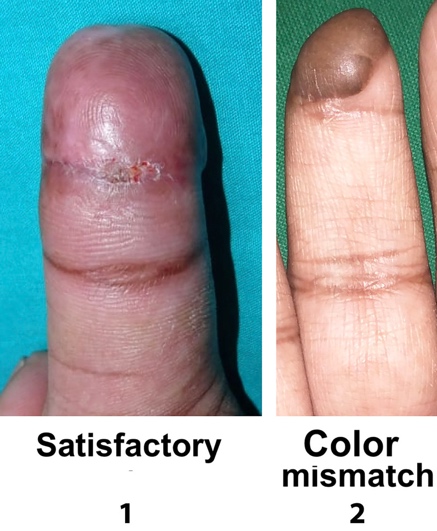


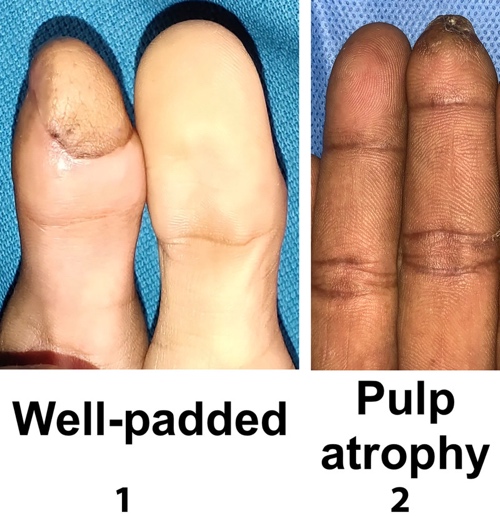

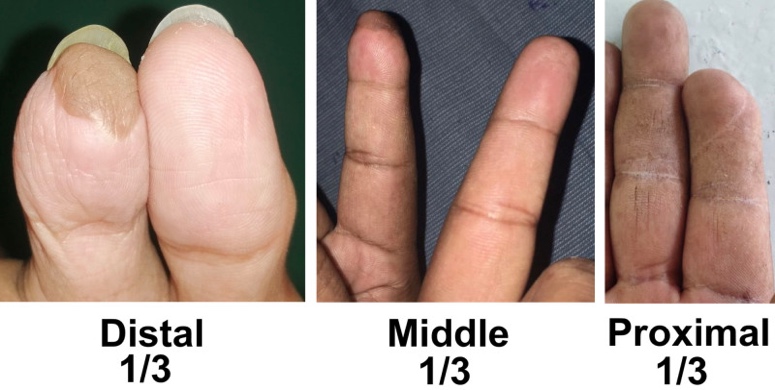


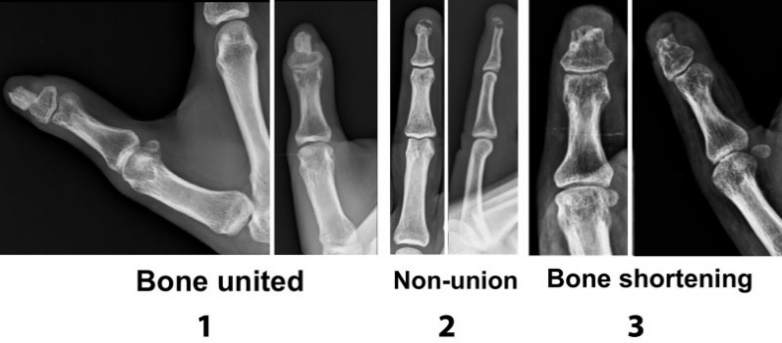


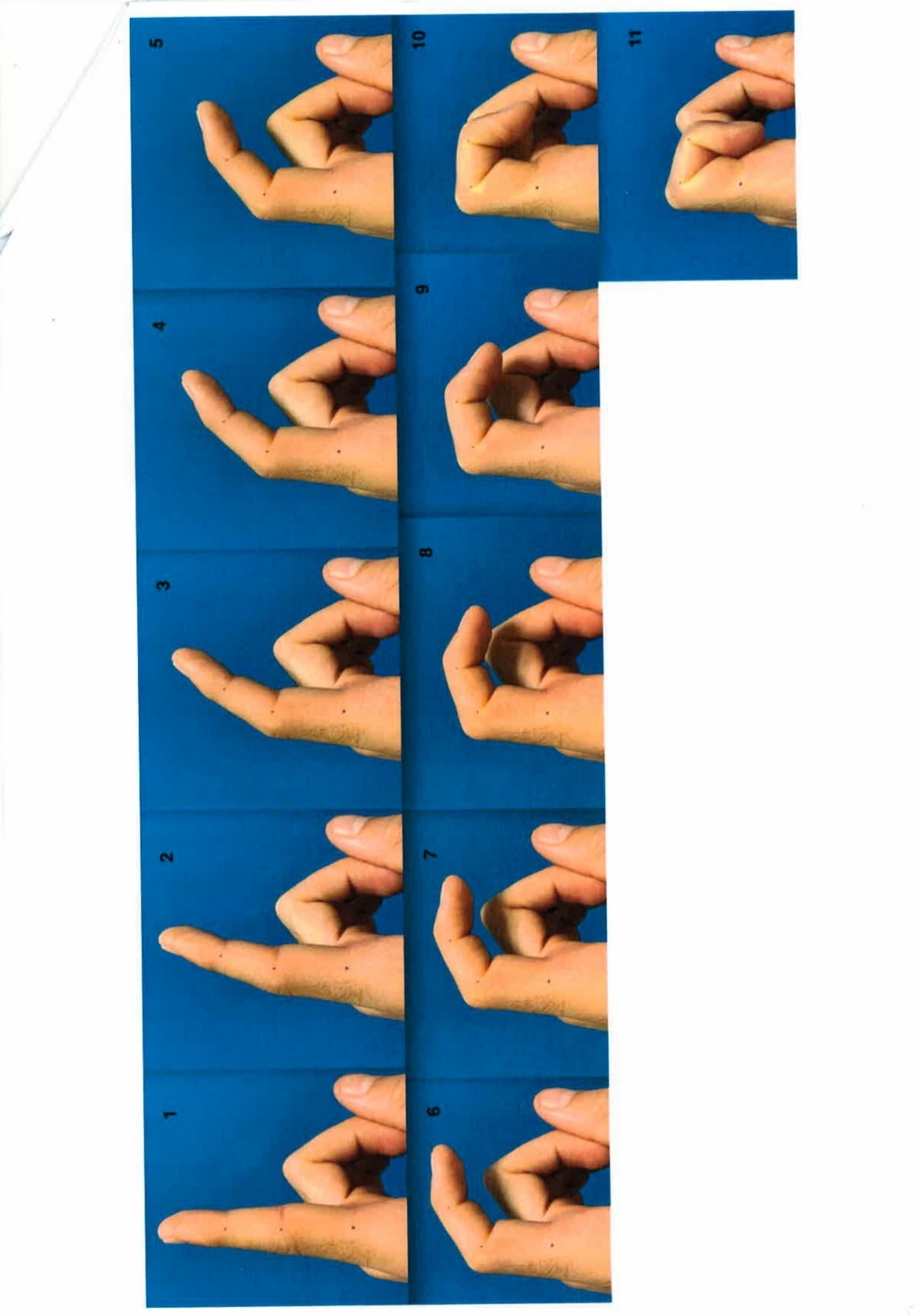
**Appendix 3: Assessment of Digital Range of Motion**
